# Metal Nanoclusters Synthesized in Alkaline Ethylene Glycol: Mechanism and Application

**DOI:** 10.3390/nano13030565

**Published:** 2023-01-30

**Authors:** Yuan Wang, Menggeng Hao

**Affiliations:** 1 Beijing National Laboratory for Molecular Sciences, College of Chemistry and Molecular Engineering, Peking University, Beijing 100871, China; 2 Sunan Institute for Molecular Engineering, Peking University, Changshu 215500, China

**Keywords:** metal nanocluster, metal catalyst, sensor, formation mechanism, unprotected metal nanoclusters

## Abstract

The “unprotected” metal and alloy nanoclusters (UMCs) prepared by the alkaline ethylene glycol method, which are stabilized with simple ions and solvent molecules, have the advantages of a small particle size, a narrow size distribution, good stability, highly efficient preparation, easy separation, surface modification and transfer between different phases. They can be composited with diverse materials to prepare catalytic systems with controllable structures, providing an effective means of studying the different factors’ effects on the catalytic properties separately. UMCs have been widely used in the development of high-performance catalysts for a variety of functional systems. This paper will review the research progress on the formation mechanism of the unprotected metal nanoclusters, exploring the structure–function relationship of metal nanocluster catalysts and the preparation of excellent metal catalysts using the unprotected metal nanoclusters as building blocks or starting materials. A principle of the influence of carriers, ligands and modifiers in metal nanocluster catalysts on the catalytic properties is proposed.

## 1. Introduction

Metal nanoclusters refer to metal and alloy nanoparticles with a small size and a narrow size distribution. They are important elements of functional systems such as catalysts and sensors, playing important roles in the chemical industry, the development and utilization of sustainable energy, environmental protection, and basic research on catalysis science [1,2,3,4,5,6]. Many factors may influence the catalytic properties of metal nanocluster catalysts [7,8,9]. In the field of metal catalysts, it has long been a challenge to analyze the individual effects of the metal particle’s size, composition, support and modifier on the catalytic properties. This is related to another challenge in the field, namely the preparation of metal catalysts with controllable structures. In the traditional preparation methods of metal catalysts, such as the impregnation method, the abovementioned factors are often coupled with each other. To solve these problems, we proposed a solution, which is to use “unprotected” metal and alloy nanoclusters as building blocks to assemble or synthesize structurally controllable catalysts [10,11,12,13,14,15,16,17,18,19,20,21,22,23].

In 2000 [10], we reported the first “unprotected” or surfactant-free platinum group metal nanoclusters (Pt, Rh and Ru) with small particle sizes (d_av_ = 1–3 nm) which were stabilized with adsorbed simple ions such as OH^−^, Cl^−^, etc. and solvent molecules, and they were prepared by a so-called alkaline ethylene glycol method (alkaline EG method). Many unprotected colloidal metal nanoclusters of the Pt group elements and their alloys (Pt, Ru, Rh [10,24], Ir [19,25], Os, PtRh [13] and PtRu [20,26], etc.) can be prepared by using this method. 

Recently, we succeeded in the preparation of unprotected PtCu alloy nanoclusters [21]. This is an important addition to the family of unprotected alloy nanoclusters because there is a strong electronic interaction between the light transition metals and the Pt group metals, which is conducive to the regulation of the catalytic properties.

The unprotected metal nanoclusters prepared by using the alkaline EG method have the advantages of a small particle size, a narrow size distribution, good stability and highly efficient preparation. They can be separated from the preparation system by adding an aqueous solution of HCl to form a precipitate of the metal nanoclusters that could be re-dispersed in many organic solvents such as alcohol, ketone, THF, DMF and DMSO to form stable colloidal solutions without an obvious change in the metal particles’ size. The average particle size of the unprotected metal nanoclusters could be easily tuned within the range from 1.1 to 2.4 by changing the preparation conditions including the metal concentration and water content in the alkaline EG method [10]. Metal nanoclusters obtained by achieving the surface modification of the unprotected metal nanoclusters with an organic ligand such as PPh_3_, fatty amine or PVP could be transferred into some organic solvents or water to form stable colloidal solutions [10,27,28,29,30]. These special properties make them suitable for the development of many catalysts with unique properties.

Attenuated total reflectance infrared spectroscopy (ATR-IR) was used to study the species adsorbed onto the Pt and Ru unprotected metal nanoclusters prepared by using the alkaline EG method, which had been exposed to air. It was found that a small amount of CO was adsorbed on the surface of the as-prepared metal nanoclusters, and the signal intensity of the adsorbed CO in the IR spectrum of a colloidal solution of Pt nanoclusters in EG increased with the duration of the exposure to air. After being treated with an aqueous solution of HCl, the amount of CO adsorbed onto the Pt nanocluster increased obviously [24]. These experimental phenomena suggest that Pt and Ru nanoclusters could catalyze the oxidation of ethylene glycol by oxygen to produce CO adsorbed onto the unprotected Pt and Ru nanoclusters at room temperature, and the catalytic oxidation rate under an acidic condition is much faster than that under a basic condition.

Recently, Kunz and co-workers found that after drying, OH^−^-stabilized Pt nanoclusters without adsorbed CO, which were obtained by using a phase transfer method, could be dispersed in ethylene glycol to form a stable colloid solution without a change in the metal particle’s size [31]. This is an important property, because it enables unprotected metal nanoclusters to be preserved as a solid powder for a long time.

By depositing the unprotected metal and alloy nanoclusters onto different supports, and removing the adsorbed species that are different from the usual protective agents such as polymer, surfactant or strong organic ligand [32,33,34] by washing or oxidation under mild conditions, one can make the metal nanoclusters touch the surface of different supports to prepare different catalysts having the same size or composition of metal nanoclusters. This provides favorable conditions for separately studying the influence of each of the aforementioned factors on the catalytic properties. In the past two decades, this strategy has been widely applied and developed, and important achievements have been made in the preparation of structurally controllable catalysts and the exploration of the relationship between structure and catalytic performance [35,36,37,38,39,40,41,42,43,44,45,46]. Based on the unprotected metal and alloy nanoclusters, catalysts with a high activity level, high selectivity and excellent stability for a variety of important chemical reactions have been developed [14,16,22,47,48,49,50,51,52,53,54,55].

Another challenge in catalyst preparation is to obtain heterogeneous catalysts with high metal loading and high metal dispersion, which are key materials in some functional systems such as fuel cell catalytic electrodes, spacecraft attitude adjustment engines and sensors. Great efforts have been made in the preparation of such catalytic systems using the unprotected metal nanoclusters prepared by using the alkaline EG method that act as building blocks [56,57,58,59,60,61] or starting materials [23,28,62,63,64,65,66,67], in which a series of effective strategies for assembling the metal nanoclusters on different supports have been developed [18,23,68,69,70,71,72,73,74,75,76,77,78,79,80,81].

In the last two decades, the unprotected metal nanocluster formation mechanism in the alkaline EG method was studied by different groups [10,24,82,83,84,85,86,87,88,89,90,91,92,93,94,95,96], who came to different conclusions. A main difference between the different views is whether metal ion compound nanoparticles form first or the metal ions are reduced to zero valence metal atoms first [10,97,98,99,100,101]. Recently, the evolution of metal species in the alkaline EG method was intensively studied by using the combined technologies of in situ time-resolved X-ray absorption fine structures (XAFS) and ultraviolet-visible absorption spectroscopy (UV-vis) [102], which provided convincing evidence for the former mechanism.

In view of the fact that some studies on the preparation, stabilization mechanism and utilization of the unprotected metal nanoclusters had been reviewed [15,103], in this review, we discuss the formation mechanisms of unprotected colloidal metal nanoclusters prepared by using the alkaline EG method. Research progress in the preparation and application of supported metal nanocluster catalysts prepared using the unprotected metal and alloy nanoclusters that act as building blocks or starting materials and the influences of different factors on the catalytic properties are reviewed. A principle of the influence of the support, ligand and modifier in the metal nanocluster catalysts on the catalytic properties is proposed. Finally, we present some perspectives for the development of metal catalysts based on the unprotected metal nanoclusters. We hope that this article will be helpful for readers to understand the latest progress and research status of the related fields, to use unprotected metal nanoclusters to develop high-performance functional materials and to explore or understand their structure–function relationships.

## 2. Formation Mechanism of Unprotected Nanoclusters

In a typical experiment for the preparation of unprotected metal nanoclusters by using the alkaline EG method, the pH of a glycol solution of a metal compound such as H_2_PtCl_6_·6H_2_O, RhCl_3_·3H_2_O or RuCl_3_·3H_2_O was adjusted to a value that is higher than 12 by adding a glycol or water solution of NaOH at room temperature. Then, the mixture was heated at a temperature in the range from 80 to 198 °C, with a N_2_ flow passing through the reaction system to carry away the water and by-products to produce a stable colloidal solution of unprotected metal clusters stabilized with simple ions and solvent molecules with an average diameter of 1.1–2.4 nm [10,102].

The original motivation for establishing the alkaline EG method was to make the colloidal nucleation process precede the metal ion reduction process, so as to overcome the difficultly in controlling the aggregation process of the metal atoms in the absence of usual protective agents, which usually leads to the formation of large colloidal metal particles (8–200 nm) [104,105,106].

In the early experiments on the preparation of the unprotected Pt nanoclusters, it was found that there were intermediates of nanoparticles of non-zero-valent platinum compounds in the reaction systems [10,84,107], which is evidence of the nucleation reduction step-by-step formation mechanism of the unprotected metal nanoclusters.

**Figure 1:**
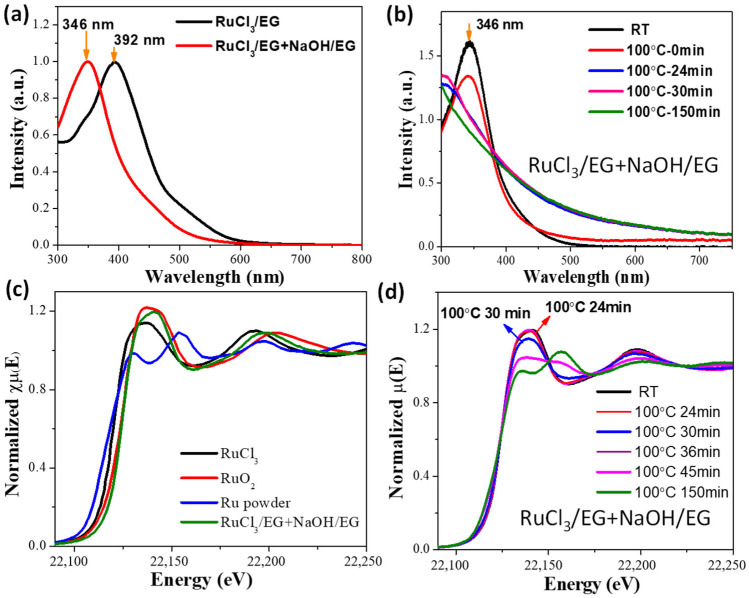
In situ characterization of colloidal nanoclusters. (**a**) UV-vis absorption spectra of RuCl_3_ glycol solution and RuCl_3_-NaOH/EG solution; (**b**) the in situ UV-vis absorption spectra of reaction mixture for preparing unprotected Ru nanoclusters under different conditions; (**c**) Ru *K*-edge XANES spectra of Ru powder, RuCl_3_ glycol solution and RuO_2_ powder; (**d**) time evolution of Ru *K*-edge XANES spectra of reaction mixture for preparing unprotected Ru nanocluster under different conditions. Reproduced with permission, Copyright 2020 Editorial Office of Acta Physico-Chimica Sinica [102].

**Figure 2:**
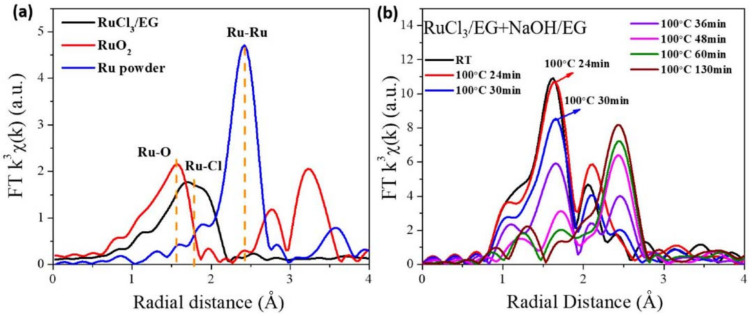
Fourier transforms of k^3^-weighted Ru *K*-edge EXAFS spectra (radial distribution function). (**a**) The reference of Ru powder, RuCl_3_ glycol solution and RuO_2_ powder; (**b**) the reaction mixture for preparing unprotected Ru nanoclusters under different conditions. Reproduced with permission, Copyright 2020 Editorial Office of Acta Physico-Chimica Sinica [102].

Recently, we studied the evolution of Ru or Pt species in the alkaline EG method by performing in situ time-resolved X-ray absorption fine structure (XAFS) and in situ UV-vis absorption spectroscopy, meanwhile, the sizes of nanoparticles formed in the reaction system were analyzed by performing TEM and dynamic light scattering (DLS) [102]. Figure 1 shows the UV-vis absorption spectra and time-resolved Ru *K*-edge X-ray absorption near-edge structure (XANES) spectra of the reaction mixtures in the preparation of unprotected Ru nanoclusters under different conditions, and the parameters of the Ru–Cl, Ru–O, and Ru–Ru bonds acquired from the extended X-ray absorption fine structure (EXAFS) analysis (Figure 2) are listed in Table 1. These data demonstrated that the substitution of Cl^−^ in RuCl_3_ by OH^−^ to form [Ru(OH)_6_]^3−^ occurred in a temperature that was lower than 45 °C. After 24 min of reaction at 100 °C, the Ru oxide nanoparticles formed as shown in the UV-vis absorption spectrum with strong light scatting signals of formed nanoparticles in the reaction mixture, while at this time, Ru in a metallic state or the Ru-Ru bond had not yet formed (see Table 1). As the reaction progressed, the coordination number (CN) of Ru–O gradually decreased. After 30 min of reaction at 100 °C, the formation of Ru–Ru bonds was observed, after which the Ru metal nanoclusters formed rapidly.

**Figure 3:**
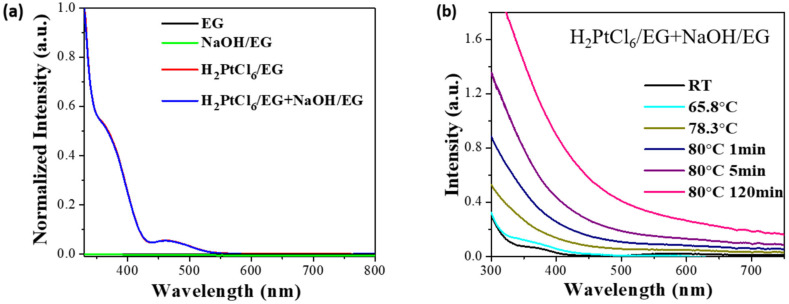
UV-vis absorption spectra. (**a**) The absorption spectra of EG, H_2_PtCl_6_/EG, NaOH/EG and the mixed solution of H_2_PtCl_6_/EG and NaOH/EG. (**b**) The in situ absorption spectra of reaction mixture for preparing unprotected Pt nanoclusters during reduction. Reproduced with permission, Copyright 2020 Editorial Office of Acta Physico-Chimica Sinica [102].

**Figure 4:**
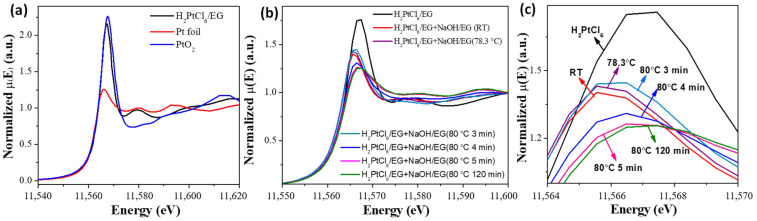
Pt *L*_3_-edge XANES spectra. (**a**) The reference of Pt foil, H_2_PtCl_6_ glycol solution and PtO_2_ powder; (**b**) time evolution of reaction mixture for preparing Pt nanoclusters; (**c**) the enlarged image of the absorption peak at ca. 11565 eV. Reproduced with permission, Copyright 2020 Editorial Office of Acta Physico-Chimica Sinica [102].

**Figure 5:**
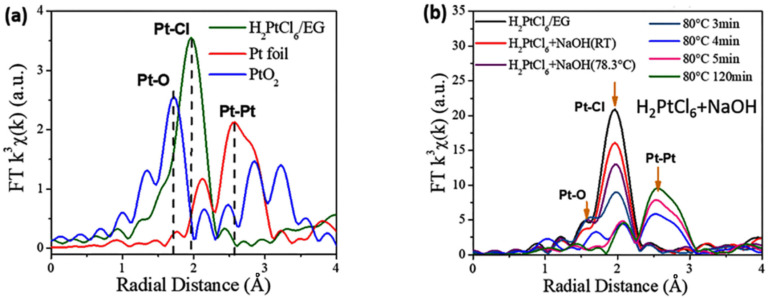
Fourier transforms of *k*^3^-weighted Pt *L*_3_-edge EXAFS spectra (radial distribution function). (**a**) The reference of Pt foil, glycol solution of H_2_PtCl_6_ and PtO_2_; (**b**) the reaction mixture for preparing unprotected Pt nanoclusters under different conditions. Reproduced with permission, Copyright 2020 Editorial Office of Acta Physico-Chimica Sinica [102].

The in situ UV-vis absorption spectra and Pt *L*_3_-edge XANES spectra of the reaction mixtures in the preparation of unprotected Pt nanoclusters under different conditions are shown in Figure 3 and Figure 4, respectively. The results indicated that during the synthesis of unprotected Pt nanocluster colloids, a part of Pt (Ⅳ) was reduced to Pt (Ⅱ) in the alkaline EG at room temperature. During the heating process from room temperature to 80 °C, Cl^−^ coordinated with Pt ions was gradually substituted by OH^−^ (Table 2). Before the formation of the Pt–Pt bond at 80 °C was detected by XAFS (Figure 5), clear-cut nanoparticle scattering signals were observed in the UV-vis absorption spectra of the reaction system at 78.3 °C (Figure 3b), suggesting that the Pt oxide or hydroxide nanoparticles had formed. As the reaction progressed at 80 ℃, the Pt–Pt bond formed in the reaction system, and the signals of the Pt–O and Pt–Cl bonds detected by XAFS gradually disappeared, corresponding to the formation of unprotected Pt nanoclusters by the reduction of Pt oxide nanoparticles. The average diameter of the nanoparticles formed in the reaction system for preparing Pt NCs were analyzed by TEM and DLS (Table 3). These results indicated that the products of the reaction of the Pt (Ⅳ) and Pt (Ⅱ) species with OH^−^ were further condensed to form oxide or complex nanoparticles, which finally were reduced to Pt nanoclusters by EG or the organic intermediates formed during the process.

**Figure 6:**
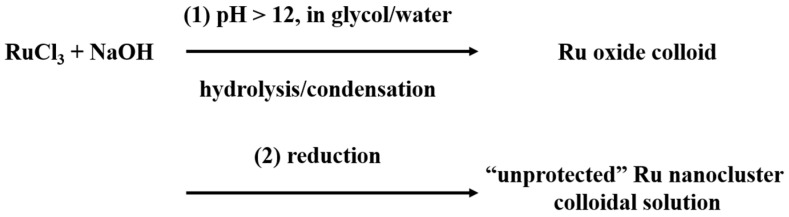
Procedure of the alkaline EG method for the preparation of unprotected Ru nanoclusters.

The aforementioned in situ time-resolved experimental results that were obtained at relative low temperatures prove the nucleation reduction step-by-step mechanism, as illustrated in Figure 6, which explains why the alkaline EG method can efficiently prepare unprotected, small metal nanoclusters in solutions with high metal concentrations [102].

## 3. Applications of “Unprotected” Metal Nanoclusters

### 3.1. Application in Catalysis

#### 3.1.1. Exploring the Structure–Function Relationship of Metal Nanocluster Catalysts

It is well known that the composition, size and surface structure of metal nanoclusters, as well as the environment surrounding the metal nanoclusters, have significant influences on the electronic and geometric structures of the metal nanoclusters, which determine the catalytic activity, selectivity and stability of the metal nanocluster-based catalysts [108,109,110,111]. Unprotected metal nanoclusters provide tractable tools for experimentally studying the effects of individual factors such as the metal composition, size, support, and the surface modification groups on the catalytic properties of metal nanocluster catalysts by assembling structure-controllable catalysts with unprotected metal or alloy nanoclusters that act as the building blocks [21,45,112,113,114,115]. A theoretical calculation based on the experimental results could further deepen the understanding of the relationship between the structure and performance of the catalytic sites of metal nanocluster catalysts [55,116]. Based on the relevant experimental and theoretical research results described in this section, in this paper, we propose a principle of the influence of carriers, ligands and modifiers in metal nanocluster catalysts on the catalytic properties. The main points of the principle are as follows:

Chemical bonding or electron transfer between carriers, ligands or modifiers and metal nanoclusters can alter the distribution of electrons (or charge) and the metal atom spacing of the metal nanoclusters in the catalysts. The changes have the following characteristics:

(1) They change the extent of charge separation between the surface atomic layer and core of the metal nanocluster. The electron donation from the support or ligand will increase the charge separation extent, leaving the surface atomic layer with a more negative charge.
(2) They change the charge distribution state of the metal nanocluster surface atomic layer at the atomic (or subatomic) scale and make it more uneven compared with that of the naked metal nanocluster.
(3) They change the distance between some of the metal atoms.
(4) The extent of the change in the charge distribution or atomic spacing is related to the size of the metal nanocluster. Usually, small-sized metal nanoclusters exhibit more significant changes.
(5) Catalytic sites composed of surface metal atoms with different charge states or atomic spacings exhibit different adsorption energies for a reactant and reaction energy barrier, showing different catalytic activities and product selectivities. The species in the support surface (the ion, vacancy or chemical group) or the ligand adjacent to the metal nanocluster can form complex (or synergistic) catalytic sites with metal nanocluster surface atoms, at which the adsorption energies of the reactants and chemical reaction energy barriers are influenced by coordination polarization or hydrogen bonding, resulting in a synergistic catalytic effect.

**Figure 7:**
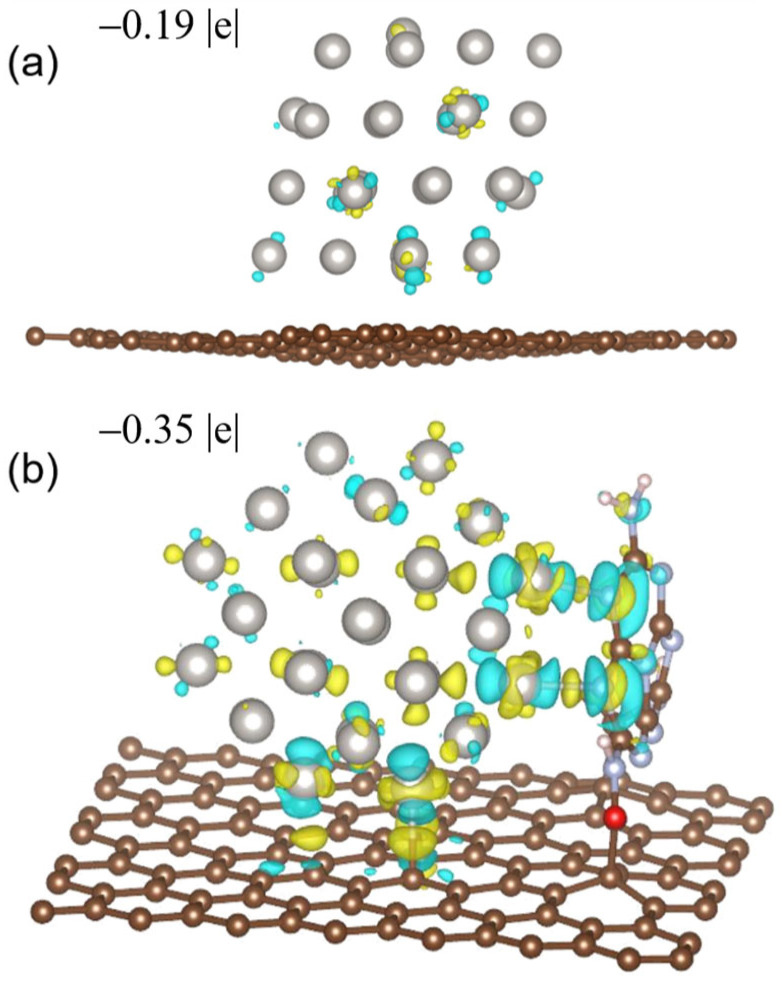
The charge density difference (isosurface unit = 4 × 10^−3^ e per Bohr^3^) and Bader charges of Pt_40_ NCs of (**a**) Pt_40_/C and (**b**) Pt_40_/MMC. The yellow and blue surface represent the charge increase and decrease, respectively. The Bader charges of the Pt atoms at the marked adsorption sites for OOH* and OH^−^* on Pt/C with an average value of −0.049 |e| are less than that on Pt/MMC (with an average value of −0.077 |e|). Reproduced with permission, Copyright 2022 Wiley-VCH GmbH [116].

**Figure 8:**
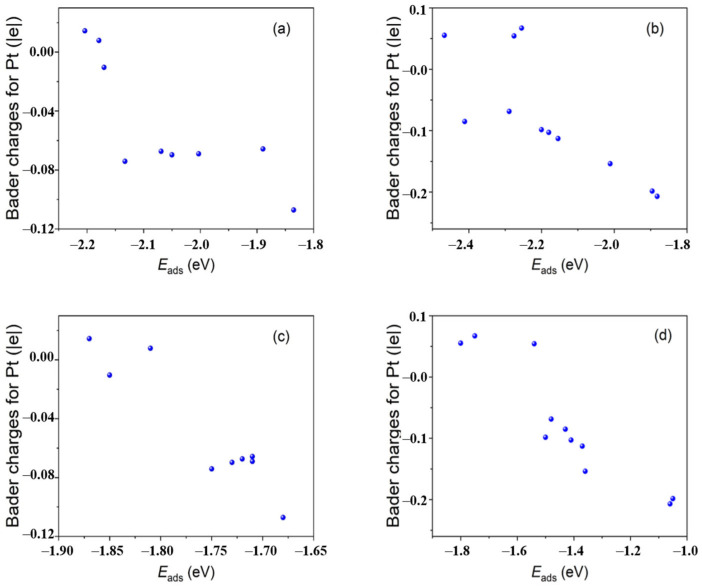
Plots of Bader charges of the representative adsorption site Pt atoms versus adsorption energies of OOH* on Pt_40_ NCs of Pt_40_/C (**a**) and Pt_40_/MMC (**b**); plots of Bader charges of the representative adsorption site Pt atoms versus adsorption energies of OH^−^* on Pt_40_ NCs of Pt_40_/C (**c**) and Pt_40_/MMC (**d**). Reproduced with permission, Copyright 2022 Wiley-VCH GmbH [116].

In a recent research [116], we synthesized two catalysts (Pt/MMC-6.8N and Pt/VXC-72R) with the same sized Pt nanoclusters and different supports by assembling the unprotected Pt nanoclusters with a mean diameter of 1.4 nm with a melem-modified carbon carrier containing 6.8 wt% of N (MMC-6.8 N) and a carbon carrier VXC-72R. These catalysts were used to catalyzed the oxygen reduction reaction (ORR), a crucial reaction in fuel cells. It was found that the mass activity (MA) of Pt/MMC-6.8N at 0.9 V (vs. RHE) reached 907 mA mg^−1^_Pt_ (this is 2.1 times larger than the U.S. Department of Energy’s target for 2020 (440 mA mg^−1^_Pt_)), which was 3.9 and 4.2 times larger those of Pt/VXC-72R and a commercial Pt/C catalyst of similar Pt particle sizes, respectively. After ten thousand voltage cycles from 1.0 to 1.5 V at 60 °C, the MA of Pt/MMC-6.8N decreased by 2%, while the MA loss of Pt/VXC-72R was 40%. The remarkable promotion effects of MMC on the catalytic activity and durability were intensively studied using a series of characterization technologies and a density functional theory (DFT) calculation. X-ray photoelectron spectroscopy (XPS) and XANES are commonly used to investigate the relationship between the catalytic performance and electron binding energy or electron occupation status of metal nanocluster catalysts. To our surprise, we could not find any obvious difference in the apparent valence states of the Pt nanoclusters between the two catalysts in the XPS and XANES analyses. However, the DFT calculation based on the model catalysts for the two prepared catalysts demonstrated obvious differences in the charge distribution on the metal nanocluster surfaces and across the entire metal nanoclusters. The model catalysts for Pt/VXC-72R and Pt/MMC-6.8N (Pt_40_/C and Pt_40_/MMC) are composed of Pt NCs with 40 Pt atoms (Pt_40_ NCs, about 1 nm in diameter) and a graphene sheet and a melem-modified graphene sheet, respectively. It was found that the Pt_40_ NC in Pt_40_/MMC is chemically bounded to two nitrogen atoms of the melem group and one C atom of the melem-modified graphene sheet, while the Pt_40_ NC in Pt_40_/C does not form any chemical bond with the graphene sheet. The charge density differences and Bader charges of Pt_40_ NCs in Pt_40_/C and Pt_40_/MMC have been calculated, and they are shown in Figure 7. It was found that the Bader charges of Pt_40_ NCs in Pt_40_/MMC (−0.35 |e|) are close to those in Pt_40_/C (−0.19 |e|), which is consistent with the XANES and XPS characterization results, reflecting the average electronic states of the Pt NCs in the catalysts. However, the negative charge of the outer layer Pt atoms in Pt_40_ NC of Pt_40_/MMC is much larger than that of Pt_40_/C, and the charging extents of different surface atoms of Pt_40_ NCs in Pt_40_/MMC are quite different, which are derived from the interaction between MMC and Pt_40_ NC (Figure 8).

The high negative charge of the catalytic site weakens the adsorption of OH^−^*. On the other hand, the formation of a hydrogen bond between the intermediate OOH* and N atoms in MMC on some catalytic sites of Pt_40_/MMC increases the adsorption energy of OOH*. The obviously enhanced catalytic activity for ORR of Pt/MMC compared that of Pt/C is attributed to the decrease in OH^−^* adsorption energy and the increase in OOH* adsorption energy at some sites of Pt/MMC. The chemical bonds between the Pt nanoclusters and the MMC carriers explain the high durability of Pt/MMC.

In 2004, we were the first to present that compared with the catalytic sites of the metal nanocluster themselves, the catalytic sites composed of metal nanocluster surface atoms and the adjacent surface oxygen vacancy of the metal oxide supports may exhibit much better catalytic properties [11], indicating a synergistic effect between the metal nanoclusters and adjacent surface oxygen vacancies. For a Ru/SnO_2_ catalyst assembled with unprotected Ru nanoclusters and tin oxide colloidal nanoparticles, with an average Ru particle size of 1.3 nm, the average catalytic activity (4.3 × 10^−2^ mol*_o_*_-CNB_ mol^−1^_Ru_ s^−1^) for the selective hydrogenation of ortho-chloronitrobenzene (*o*-CNB) to ortho-chloroaniline (*o*-CAN) was much higher than that of the PVP-protected Ru nanoclusters prepared with the same unprotected Ru nanoclusters (6.9 × 10^−3^ mol*_o_*_-CNB_ mol^−1^_Ru_ s^−1^). It was proposed that the oxygen vacancies or coordination-unsaturated Sn^4+^ or Sn^2+^ species at the support surface surrounding the Ru metal nanoclusters may activate the polar NO_2_ groups of *o*-CNB and coordinate with the NH_2_ groups of the produced *o*-CAN molecules, thereby significantly promoting the hydrogenation of *o*-CNB and depressing the dehalogenation of *o*-CAN. The selectivity for *o*-CAN of Ru/SnO_2_ reached 99.9%, which is much higher than that of the Ru/SiO_2_ prepared with the same unprotected Ru nanoclusters (95%) after complete *o*-CNB conversion, which is due to the hydrodechlorination rate being much lower for Ru/SnO_2_. Soon after, this concept was successfully applied to develop highly efficient catalysts for the selective hydrogenation of halonitrobenzenes to corresponding haloanilines by depositing unprotected Pt metal nanoclusters onto iron oxide supports [12,16,19]. It was found that during the activation of the catalysts, Pt nanoclusters could catalyze the partial reduction of iron oxide, thus forming oxygen vacancies surrounding the Pt nanoclusters. The partially reduced iron-oxide supported Pt nanocluster catalyst showed much high catalytic activity and selectivity for *o*-CAN than the Pt/C catalyst with the same size of Pt nanoclusters did, and the catalytic dehalogenation side reaction of was completely suppressed at 100% conversion of the substrates over the Pt/iron oxide catalysts. The interaction between the Pt nanoclusters and the surface oxygen vacancies resulted in an increase in the electron binding energy of Pt 4f_7/2_ in the partially reduced Pt/γ-Fe_2_O_3_ (Pt/γ-Fe_2_O_3_-PR), which is 0.5 eV higher than that of the same sized Pt nanoclusters protected by PVP, as measured using an in situ XPS spectrometer, indicating the electron transfer from Pt nanoclusters to iron cations in the catalyst [16]. The high selectivity for haloanilines in the hydrogenation of halonitrobenzenes was attributed to the electron-deficient state of the Pt nanoclusters supported on the partially reduced iron oxide supports, which may have weakened the extent of electron feedback from the Pt particles to the aromatic ring in *o*-CAN and suppressed the hydrodechlorination of the haloanilines.

Pt/γ-Fe_2_O_3_, Pt/α-Fe_2_O_3_, Ir/γ-Fe_2_O_3_, Rh/γ-Fe_2_O_3_ and Ru/γ-Fe_2_O_3_ were prepared by depositing the corresponding unprotected platinum group metal (PGM) NCs onto the iron oxide supports and treating the solid products with H_2_ at 373 K. A universal rule was revealed for the first time through IR-CO probe and CO chemisorption measurements on these samples, namely, the PGM NCs supported on partially reduced iron oxides have an extremely weak affinity for CO [16,19]. This should be derived from the electron transfer from PGMs NCs to the surface oxygen vacancies of the iron oxides and weaken the degree of electron back-donation from the nanocluster surface atoms to CO. This discovery is very helpful for understanding the unique catalytic properties of these catalysts in many reactions.

Recently, Deng and co-workers prepared different catalysts (Pt/CoNi@NC, Pt/CNT and Pt/C) with the same Pt loading (4 wt%) by loading the same unprotected Pt nanoclusters (1–2 nm) onto graphene-encapsulated CoNi alloy NCs, commercial carbon nanotube (CNT) and carbon black (CB), respectively. The Pt nanoclusters isolated by graphene from CoNi nanoparticles in Pt/CoNi@NC exhibited much higher catalytic activity than Pt/CNT and Pt/C did for the oxidation of CO, with a 100% conversion of CO at room temperature in an oxygen-rich atmosphere. The high catalytic activity was attributed to the electron penetration effect, which makes the Pt-graphene interface perform catalytic activity for CO oxidation, providing a new idea for regulating the electronic structure of metal nanocluster catalysts [117].

We succeeded in the preparation of unprotected PtCu alloy nanoclusters with solid solution structures and controllable Pt-to-Cu ratio (1:3-9:1) using a modified alkaline glycol method by introducing acetate ions into the reaction system [21,118]. MMC-supported alloy nanocluster catalysts (PtCu/MMC) with an average diameter of ca. 2 nm and different Pt/Cu ratios were prepared by assembling the PtCu alloy nanoclusters and MMC, which were used as catalysts for ORR. The alloy catalyst with a Pt/Cu ratio of 3:1 showed not only the highest MA (1.59A mg^−1^_Pt_ @0.9 V), but it also showed the highest specific activity (SA) (3.98 mA cm^−2^_Pt_ @0.9 V) among the tested catalysts. As the particle sizes of the alloy nanoclusters in the catalysts are almost the same, the effect of the Pt/Cu ratio on the activity should be mainly derived from the electronic properties of the nanoclusters. As revealed by the in situ XPS measurements, the electron transfer from Cu to Pt occurred in the alloy nanoclusters, which may be the reason why PtCu alloy catalysts exhibit enhanced catalytic activity for ORR compared to that of Pt/MMC. From a further comparison of the catalytic activation of Pt_3_Cu_1_/MMC, Pt_3_Cu_1_/C and Pt/C, it was found that the alloying effect increased the MA by 1.4 times, while the promoting effect of MMC increased the MA by 3.2 times.

We prepared a PtRu/NCNHs composite using N-doped carbon nanohorns (NCNHs) and unprotected PtRu NCs with an average PtRu particle size of 1.9 nm, which was used as a catalyst for the electrochemical oxidation of methanol [20]. The MA of PtRu/NCNHs (850 mA mg^−1^_PtRu_ @0.9 V) was 2.5 and 1.7 times larger than those of a commercial PtRu/C catalyst and a homemade PtRu/Vulcan carbon catalyst, respectively. The SA of PtRu/NCNHs (1.35 mA cm^−2^_PtRu_ @0.9 V) was 1.8 times larger than that of PtRu/Vulcan carbon. The TEM, ICP and XPS results showed that the three catalysts had similar PtRu particle sizes and Pt/Ru atomic ratios. The Pt 4f and Ru 3p electron binding energies of PtRu/NCNHs had a negative shift of 0.2 eV compared with the corresponding values of PtRu/Vulcan, which was attributed to metal–support interactions, indicating the electron transfer from NCNHs to PtRu NCs in the PtRu/NCNHs catalyst. Based on the proposed principle described above, the improved catalytic activity for the oxidation of CH_3_OH and carbonaceous intermediates of PtRu/NCNHs compared to that of PtRu/Vulcan carbon might be derived from the unique structure of complex catalytic sites in the catalyst.

In order to reveal the ligand effects, we investigated the effect of the organic ligands on the Pt 4f electron binding energies of small Pt nanoclusters [27]. The same sized Pt nanoclusters (d_av_ = 1.3 nm, size distribution of between 0.8 and 2.8 nm) modified with C_12_H_25_NH_2_, C_12_H_25_SH, PPh_3_, polyvinylpyrrolidone (PVP) or polyvinyl alcohol (PVA) were prepared by the surface modification of the unprotected Pt nanoclusters. The 4f_7/2_ level electron binding energies of Pt in the prepared C_12_H_25_NH_2_-, PVP-, PVA-, PPh_3_- and C_12_H_25_SH-protected Pt nanoclusters increased by 0.5, 0.5, 0.5, 0.6 and 0.8 eV than that of bulk Pt, respectively, as measured by XPS. Since the weak interaction between the PVA and Pt nanoclusters cannot affect the core-level electron binding energy of the Pt core to an observable extent, the increment in the Pt 4f binding energy of the PVA-protected Pt nanocluster was mainly derived from the metal particle size effect, originating from the final state relaxation [27]. The surface modification of the Pt nanoclusters with C_12_H_25_SH caused a further increase in the Pt 4f_7/2_ electron binding energies of 0.3 eV, which is mainly caused by the formation of the Pt–S bond and the coordination of mercaptan groups on the surface Pt atoms. Although the measured Pt 4f electron binding energies of the Pt nanoclusters protected by C_12_H_25_NH_2_, PVP, PVA or PPh_3_ are very similar to each other, the charge distribution of these metal nanoclusters should be quite different since the intensities of electronic interaction between these ligands and Pt nanoclusters are different. Recently, the catalytic activities of these protected Pt nanoclusters for the hydrogenation of para-chloronitrobenzene (*p*-CNB) were measured [45]. The initial catalytic activities of the C_12_H_25_SH-, PPh_3_-, C_18_H_37_NH_2_- or PVP-modified Pt nanoclusters with the same sized Pt nanoclusters were 4.7, 4.1, 3.1 and 1.6 mol_hydrogen_ (mol_Pt_ S)^−1^, respectively, and the selectivity for the byproduct aniline at a *p*-CNB conversion of about 40% followed the trend: Pt–PPh_3_ > Pt–C_18_H_37_NH_2_ > Pt–PVP > Pt–C_12_H_25_SH. We believe that the activity and selectivity of these protected Pt NCs are related to the different charge distribution of the metal NCs.

In recent years, the unprotected platinum group metal and their alloy nanoclusters prepared by using the alkaline EG method have been widely used as building blocks for fabricating excellent organic ligands-modified metal or alloy nanocluster catalysts for the hydrogenation of aromatic chloronitro compounds, asymmetric hydrogenation of keto esters or hydrogenation of 3-hexyne, etc. [119,120,121,122,123], and the effect of the ligands on the catalytic properties of metal or alloy nanocluster catalysts were studied [35,38,95,124,125,126,127,128,129,130].

The size effects of Pt nanoclusters in Pt/Fe_2_O_3_ catalysts for the CO oxidation at room-temperature were investigated by Zhang et al. [131]. The Pt/Fe_2_O_3_-a, Pt/Fe_2_O_3_-b and Pt/Fe_2_O_3_-c catalysts were prepared by depositing unprotected Pt nanoclusters with mean diameters of 1.1, 1.9 and 2.7 nm on the surface of Fe(OH)_3_ powders, respectively, followed by high-temperature calcination in a flow of 20% O_2_/Ar. Unprotected Pt nanoclusters colloids with different average particle sizes were prepared by changing the metal concentration or water content in the alkaline EG method [10,131]. The catalytic tests on the oxidation of CO to CO_2_ at a low temperature showed that the Pt/Fe_2_O_3_-b catalyst exhibited the highest activity among the tested catalysts, with a 42% conversion of CO at room temperature and a complete conversion of CO at around 60 ℃. For Pt/Fe_2_O_3_-a and Pt/Fe_2_O_3_-c, the CO conversion rates were 20% and 8% at room temperature, while the complete conversion of CO was realized at 70 and 90 ℃, respectively. The XPS and XANES measurement results indicate that the Pt nanocluster size affected the Pt species chemical states in the Pt/Fe_2_O_3_ catalysts. The Pt atoms in Pt/Fe_2_O_3_-a had been largely oxidized to Pt^2+^, while the majority of the Pt atoms in Pt/Fe_2_O_3_-b and Pt/Fe_2_O_3_-c were in the Pt^0^ oxidation state, with Pt/Fe_2_O_3_-c containing the most Pt atoms in a metallic state.

#### 3.1.2. Application in Fabrication of Smart Catalysts

The unprotected metal nanoclusters prepared based on the alkaline EG method have been widely applied in the fabrication of metal nanocluster-based catalysts with excellent catalytic properties [53,54,55,132,133,134,135,136,137,138,139,140,141].

Size- or shape-selective catalysts have important applications for the production of fine chemicals. Li and Yang et al. reported the first example of the encapsulation of unprotected Pt nanoclusters in zeolitic imidazolate frameworks (ZIFs) to prepare heterogeneous catalysts with high size selectivity for the substrates in the catalytic hydrogenation of alkenes [50]. In this preparation, 2-Methyl imidazole plays both the roles of the Pt NPs stabilizer and the conjunction linker of ZIF-8. The catalytic performance of the Pt@ZIF-8 catalysts prepared by this hetero-nucleation strategy was much better than that of the ZIF-8 encapsulated with PVP-protected Pt NPs. It would be expected that other polydentate ligands that are capable of capping the unprotected metal NPs could also be employed to fabricate various metal NPs@MOFs with the same strategy, providing an effective way to design and synthesize size- or shape-selective catalysts. Moreover, Li and Yang et al. reported an efficient approach for the encapsulation of the unprotected Pt nanoclusters into nanocages of cage-like mesoporous silicas (CMS) [142]. The prepared Pt/CMS catalysts exhibited high selectivity (>99%) for the corresponding CAN in the hydrogenation of CNB.

Liu et al. prepared the Cu single-atom-covered Pt nanoparticles supported on carbon (Pt/C–Cu) by using carbon-supported unprotected Pt nanoclusters as starting materials. The prepared bimetallic catalysts were used for the selective hydrogenation of phenylacetylene (PA) to styrene (ST), which is a very challenging catalytic process [54]. The catalytic experiment results revealed that the selectivity of ST strongly depends on the coverage of Cu single atoms on Pt nanoparticles in the Pt/C–Cu catalysts. The selectivity of ST reached 94.4% at a complete PA conversion in the selective hydrogenation of PA over a Pt/C–Cu catalyst with an optimized Cu/Pt ratio.

**Figure 9:**
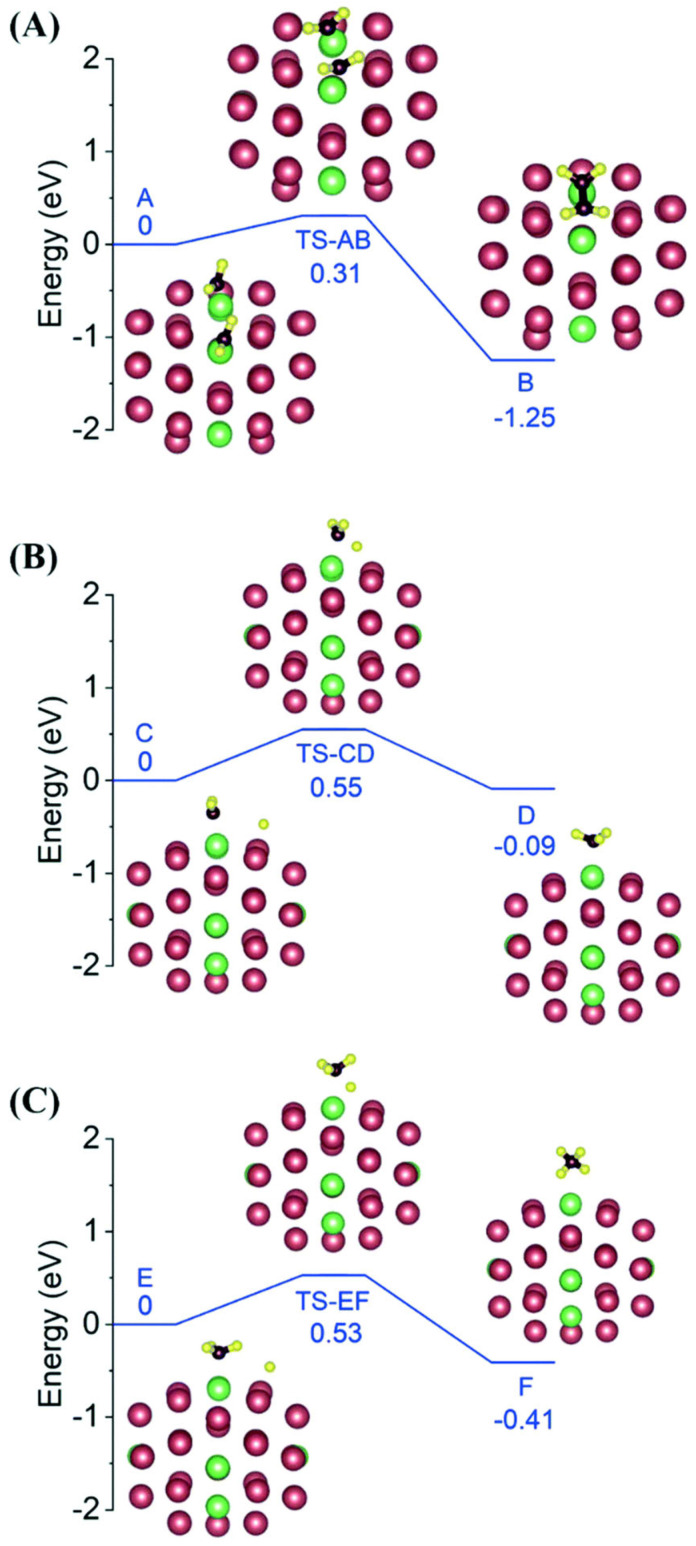
The optimized geometries of reactants, transition states and products for (**A**) CH_2_ + CH_2_ coupling, (**B**) CH_2_ hydrogenation and (**C**) CH_3_ hydrogenation on a Pt_42_-Ru_8_ bimetallic cluster, respectively. Reproduced with permission, Copyright 2022 The Royal Society of Chemistry [55].

The catalytic hydrogenation of carbon dioxide to produce multi-carbon compounds is of significance because it will make carbon dioxide an important carbon resource for the synthesis of sustainable energy sources or fine chemicals, thereby reducing the dependence on fossil fuels [143,144,145,146,147,148,149,150,151]. For most reported catalysts, the synthesis of multi-carbon compounds through CO_2_ hydrogenation usually needs to be carried out at high temperatures of 200–350 °C. The development of new catalytic systems to generate liquid fuels or highly valued fine chemicals by consuming CO_2_ under mild conditions would bring about many economic and environmental benefits. Recently, we reported a new method for the catalytic conversion of CO_2_ to multi-carbon compounds at low temperatures [53,152]. At 40–130 °C, in a catalyst of ferrous carbonate-supported Pt nanoclusters and Ru nanoclusters, the Pt and Ru nanoclusters can catalyze the hydrogenation of the carbonate ions to form high hydrocarbons and multi-carbon alcohols. The carbon atom number in the high hydrocarbons could be as high as 26. Coupling this process with the carbonation of the resulting metal species enables the catalytic conversion of CO_2_ to high hydrocarbons and multi-carbon alcohols [53]. The reaction equations are shown as follows: 
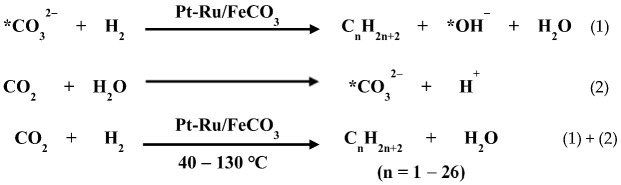


At 40 °C, the selectivities for C_5_–C_26_ hydrocarbons and C_2+_ compounds reached 49.6% and 77.7%, respectively, while at 130 °C, the selectivities for them were 26.1% and 45.8%, respectively, while for the conversion of carbon in the substrates (including FeCO_3_ and CO_2_) over 8 h, it was 5.1%. CO was not detected by gas chromatography (GC) in the products. The experimental results indicated that the simultaneous presence of platinum and ruthenium in the catalyst is beneficial to improve the selectivity of multi-carbon compounds. This encouraged us to improve the selectivity of multi-carbon compounds in the products by regulating the distribution of Pt and Ru elements in the catalyst. Pt nanocrystals anchoring small Ru clusters on carbon (Ru-*co*-Pt/C) [55] were prepared using unprotected Pt and Ru nanoclusters as starting materials through the Pt nanocluster catalyzing atomization of Ru nanoclusters and a surface growth process at 130 °C in a mixture of water and cyclohexane in a gaseous mixture of CO_2_ and H_2_ and characterized by HRTEM, EDX, EXAFS and XPS. Carbon-supported Pt or Ru nanoclusters were prepared by the immobilization of the Pt or Ru nanoclusters on a carbon support, and these were used as catalysts for comparison. At 130 °C, in a mixture of water and cyclohexane, the Ru/C catalyst exhibited a high activity for CO_2_ hydrogenation with a selectivity for methane of 94.3%, while the catalytic activity of the Pt/C catalyst was quite low under this condition. Ru-*co*-Pt/C could catalyze CO_2_ hydrogenation to produce hydrocarbons and alcohols with a selectivity of 90.1% for the C_2+_ compounds, far exceeding the previously reported values, which suggests that CO_2_ hydrogenation of Ru-*co*-Pt/C tends to produce multi-carbon compounds. The yield of organic hydrogenation products after 22 h of reaction reached 8.9% of Ru-*co*-Pt/C under this condition. Density functional theory (DFT) calculations were performed to study the origin of the significant increase in multi-carbon product selectivity in the CO_2_ hydrogenation products of the bimetallic catalyst compared to those of the Ru/C and Pt/C catalysts. The established model clusters used to simulate the three catalysts contain 50 metal atoms (Figure 9), and the bimetallic cluster has 3 Ru dimers and 2 individual Ru atoms anchored on the NC surface. The calculated reaction energy barriers of the three model catalysts are listed in Table 4.

On the Ru cluster, the energy barrier for CH_2_ + CH_2_ coupling is much higher than that for the hydrogenation of methyl group, accounting for the high selectivity for methane of the Ru nanocluster catalyst. It is interesting to find that of the Ru dimer anchored on the Pt NC surface, the CH_2_ + CH_2_ coupling energy barrier becomes much smaller than that for the hydrogenation of *CH_3_, which could be caused by the electron transfer from Ru to Pt and the change in distance between the Ru atoms compared to that in the Ru metal nanoclusters. The theoretical research results on the designed bimetallic NC Pt_42_-Ru_8_ account for the excellent catalytic selectivity to C_2+_ compounds in the hydrogenation of CO_2_ of Pt-*co*-Ru/C, and they contribute a smart catalyst structure for the CO_2_ hydrogenation to multi-carbon compounds or liquid fuels under mild conditions.

Direct alcohol fuel cells are expected to have the advantages of a high energy density and the fuel being easy to store. Sun et al. reported a highly active and stable Pt-PbO*_x_* nanocomposite catalyst (Pt-PbO*_x_* NC) for ethanol electrooxidation, which showed an onset potential that was 30 mV and 44 mV less positive, together with a peak current density that was 1.7 and 2.6 times higher than those observed over the Pt nanoparticles prepared with the unprotected Pt nanoclusters and a Pt black catalyst in the cyclic voltammogram tests, respectively. Pt-PbO*_x_* was prepared by mixing an EG solution of unprotected Pt nanoclusters and a powder of PbO_2_ nanoparticles at room temperature. It is interesting that under such a mild condition, most of the PbO_2_ nanoparticles were converted into Pb_3_O_4_ nanoparticles and a portion of lead ions were reduced to atoms in a zero-valence state, which could form bimetallic nanoclusters with the platinum nanoparticles, producing a catalyst (Pt-PbO*_x_*) composed of Pt, PbPt*_x_* and Pb_3_O_4_ nanoparticles with an average particle size of 3.23 nm [153].

Xin et al. prepared electrocatalysts (Pt/MWNT-a) by adding MWNTs to an EG solution of hexachloroplatinic acid containing 5 vol.% water, adjusting the pH of the solution to be above 13 by adding a solution of NaOH in EG (2.5 M) and heating the mixture to deposit the formed Pt NCs onto the MWNTs [68,101]. Pt/MWNT containing 10 wt.% of Pt prepared by using this method possessed well-dispersed Pt metal NCs with an average diameter of 2.6 nm and a narrow size distribution of 2–5 nm. In another MWNT-supported Pt catalyst (Pt/MWNT-b) with the same Pt content, which was prepared by using a formaldehyde reduction method, the Pt nanoparticles had an average diameter of 3.4 nm and a wide size distribution of 2–9 nm. When they were used as direct methanol fuel cell cathode catalysts, Pt/MWNT-a showed higher catalytic activity for ORR and a better cell performance than Pt/MWNT-b did.

Mao and co-worker reported a method for preparing carbon-supported noble metal catalysts with small metal NCs of 1–3 nm and high metal loadings of 30–50 wt.% [78], in which a carbon support was added into the colloidal solution of unprotected metal nanoclusters prepared by using the alkaline EG method, and the pH of the mixture was adjusted to be acidic so that metal nanoclusters could be deposited onto the support. A series of carbon-supported Pt or Pt-Ru alloy nanocluster catalysts with high metal contents were prepared by using this method. Electrocatalysts composed of different carbon carriers and small Pt NCs of ~2 nm in average size exhibited high electrochemical surface areas (ECSA) of 40–55 m^2^/g, which are much higher than those (32 m^2^/g) of a commercial E-tek Pt/C catalyst (C3-30, 30 wt.% Pt) with larger Pt nanoparticles of 3.2 nm in terms of average diameter, as measured under the same condition. Compared to the membrane electrode assembly (MEA) prepared with a commercially available E-tek Pt-Ru/C catalyst, MEAs made with the Pt-Ru/C catalysts prepared based on a modified alkaline EG process exhibited higher cell efficiencies and better stability against CO poisoning under the same test conditions.

Wan and co-workers prepared Pt/CNT composites by adding a colloidal solution of PPh_3_-modified Pt NCs in toluene [10] into a dispersion of CNT in toluene in an ultrasonic treatment, then they were treated with centrifugation and washed by toluene [30]. The PPh_3_ molecules adsorbed on the Pt NCs make it possible to transfer the Pt NCs from an ethylene glycol solution to a toluene solution, and they act as a linker between the Pt NCs and CNTs. Even the following thermal treatment process for removing PPh_3_ molecules caused some aggregation of the Pt NPs, the Pt NPs were still well dispersed on the CNTs. The prepared Pt/CNT catalyst exhibited higher catalytic activity and better anti-toxicity in the electrochemical oxidation of methanol than a commercial E-TEK catalyst did.

Behm et al. prepared four different catalysts (Pt/C/TiO*_x_*N*_y_*C*_z_*, Pt/TiO*_x_*N*_y_*C*_z_*, Pt/TiO_2_ and Pt/C) by depositing the same unprotected Pt nanoclusters onto the different support materials and treating them at 200 ℃ for 2 h under an N_2_ atmosphere [66]. The Pt NCs in the four catalysts have a mean diameter of about 1.6–1.7 nm, as measured by TEM. The influence of the supports on the catalytic properties were studied by XPS and electrochemical ORR measurements. The Pt 4f level electron binding energies of Pt/TiO_2_ and Pt/TiO*_x_*N*_y_*C*_z_* were much lower than those of Pt/C/TiO*_x_*N*_y_*C*_z_* and Pt/C. The mass activity level for ORR of Pt/TiO_2_ or Pt/TiO*_x_*N*_y_*C*_z_* was much lower than that of Pt/C and Pt/C/TiO*_x_*N*_y_*C*_z_*, and Pt/C/TiO*_x_*N*_y_*C*_z_* was more active toward ORR than Pt/C was. Moreover, the selectivity for H_2_O_2_ of Pt/C/TiO*_x_*N*_y_*C*_z_* or Pt/C was much lower than those of Pt/TiO_2_ and Pt/TiO*_x_*N*_y_*C*_z_*. The differences in the electrocatalytic properties of these catalysts were attributed to the effects of the support on the conductivity, activity and selectivity of the platinum catalysts.

Recently, we prepared a highly active In_2_O_3_-supported Pt-In alloy NC catalyst (Pt-In/In_2_O_3_, Pt content: 42 wt%) for ORR [23]. The Pt-In/In_2_O_3_ was prepared by adding an EG solution of unprotected Pt NCs (d_av_ = 1.4 nm) into a suspension of In_2_O_3_ NPs (d_av_ = 19 nm) in water, and then heating the mixture under stirring in an autoclave under a H_2_ atmosphere at 433 K. The Pt-In/In_2_O_3_ catalyst exhibited enhanced catalytic activity and durability compared to a commercial Pt/C-TKK catalyst and a Pt/In_2_O_3_ catalyst with a similar Pt content. The MA and SA of Pt-In/In_2_O_3_ were 0.32 A mg^−1^_Pt_ @0.9 V and 1.14 mA cm^−2^ @0.9 V, respectively. After an accelerated aging test of 15,000 potential cycles between 0.6 and 1.0 V, the MA loss was 6% for Pt-In/In_2_O_3_, but it was 19% for the commercial Pt/C-TKK. When the potential cycling was performed 6000 times between 1.0 and 1.6 V, the MA loss total of Pt-In/In_2_O_3_ was only 3%, while that of Pt/C-TKK was 33%. The XANES and XPS measurement results indicated the electronic transfer from In to Pt in the Pt-In alloy NCs, which was an important reason for the enhanced catalytic activity of Pt-In/In_2_O_3_ compared to that of Pt/In_2_O_3_. The high durability of the Pt-In/In_2_O_3_ catalyst was derived from the high stability of In_2_O_3_ against oxidation, the limited In leaching from the Pt-In NCs and the strong interaction between the In_2_O_3_ support and Pt-In NCs.

Xiao and co-workers prepared ZSM-5 zeolite-supported Pt nanoclusters with average particle sizes ranging from 1.3 to 2.3 nm and narrow size distributions using pre-prepared unprotected Pt nanoclusters that were 1.3, 1.5, 1.7, 1.9, 2.1 or 2.3 nm in size [154]. Catalytic tests on the oxidation of toluene as a model for volatile organic compounds (VOC) removal showed that Pt/ZSM-5 with an average Pt nanoparticle diameter of 1.9 nm has the highest activity among the tested catalysts due to a balance of Pt dispersion and Pt^0^ proportion in the catalyst. The catalyst is quite active and excellently stable, with a high tolerance to water and CO_2_.

Chen et al. prepared a series of Pt/Al_2_O_3_ catalysts with different Pt particle sizes (1.2–2.2 nm) by depositing the unprotected Pt nanoclusters produced in situ on an Al_2_O_3_ support [155]. The results showed that the catalytic activity of the Pt/Al_2_O_3_ catalysts for the oxidation of benzene increased with the decrease in the Pt particle size, which was attributed to the fact that high platinum dispersion can bring more adsorbed oxygen. The prepared Pt/Al_2_O_3_ catalyst with Pt NCs with an average diameter of 1.2 nm could catalyze the complete oxidation of benzene to H_2_O and CO_2_ at 145 ℃, and they exhibited excellent durability, not only in dry air, but also in the presence of H_2_O.

CO oxidation is of significance for both fundamental research and practical applications, including indoor air purification and CO removal from hydrogen used in fuel cells. In the past decade, important progress has been made in developing catalysts for CO oxidation at low temperatures using unprotected Pt NCs as starting materials [36,156,157,158,159]. Kunz et al. investigated the influence of Pt particle size on the catalytic properties for the CO oxidation of the Pt NCs supported on Fe_3_O_4_ [160]. In the preparation of the catalysts, colloidal solutions of Pt NCs with average diameters of 1–4 nm were prepared by modified alkaline EG methods using different Pt precursors. Zhang and co-workers prepared a series of Pt/Al_2_O_3_ and Pt/FeO*_x_* catalysts by using a “colloid-deposition method” using unprotected Pt NCs as starting materials, and the catalytic properties of these catalysts for low-temperature CO oxidation were investigated [161,162,163]. Chen et al. prepared a high efficient catalyst for CO oxidation by depositing unprotected Pt NCs pre-prepared by using the alkaline EG method on a Al_2_O_3_ support dispersed in EG with a pH value of 9 at 80 °C, followed by washing them with water, drying at 80 °C, and activating at 200 °C in a flow of H_2_/He [164]. Over this catalyst, the CO conversion reached 100% at −20 °C, and the specific catalytic activity was orders of magnitude higher than that of the currently commercially available catalysts. The catalytic site structure of the catalyst was intensively investigated and identified to be one composed of a kink site and a terrace site of Pt nanoclusters associated with OH species. The reaction of CO adsorbed on the kink sites with OH species on the terrace sites produces CO_2_, resulting in a low reaction energy barrier for CO oxidation.

Heterogeneous catalysts with active sites of a single noble metal atom (SACs) have attracted much attention due to their high noble metal utilization efficiency and excellent catalytic properties for many reactions [165,166]. The preparation of high-metal-loading and thermally stable SACs remains a challenge. Recently, Lang et al. reported a discovery that the unprotected Pt NPs with particle sizes of 2~3 nm supported on a Fe_2_O_3_ support with a 0.3~1 wt% loading could be completely transformed into atomically dispersed Pt atomic species on the iron oxide support with a very high density of isolated Pt atoms upon calcination under flowing air at 800 °C for 5 h [81]. Isolated Pt atoms with high oxidation state in the catalysts are stabilized by strong covalent interaction with the iron and oxygen atoms on the surface. The mechanism of the spontaneous dispersion phenomenon was explained by the partial oxidation of the surface of the Pt NPs, the vaporization of PtO_2_ and the strong interaction between the carrier and single Pt atomic species.

The heterogeneous metal catalyst prepared using the unprotected metal NCs as crucial materials have been applied as credible ones to study the difference in the catalytic properties for water gas shift reaction [167,168], the reduction of NO by H_2_ [169] and NO oxidation [170] between the metal nanoclusters and single metal atom species. They were also used for developing promising catalysts for oxygen evolution reactions [171,172], the oxidation of formaldehyde [173,174] and ammonia [175,176], the dehydrogenation of N-heterocycle compounds [177] and alkane [178,179], the photocatalytic selective oxidation of benzyl alcohol [180], the photocatalytic production of H_2_ [181], the photocatalytic degradation of phenol [182], Li-O_2_ battery cathodes [183,184] and dye-sensitized solar cells electrodes [185,186], etc.

### 3.2. Application in Sensor

**Figure 10:**
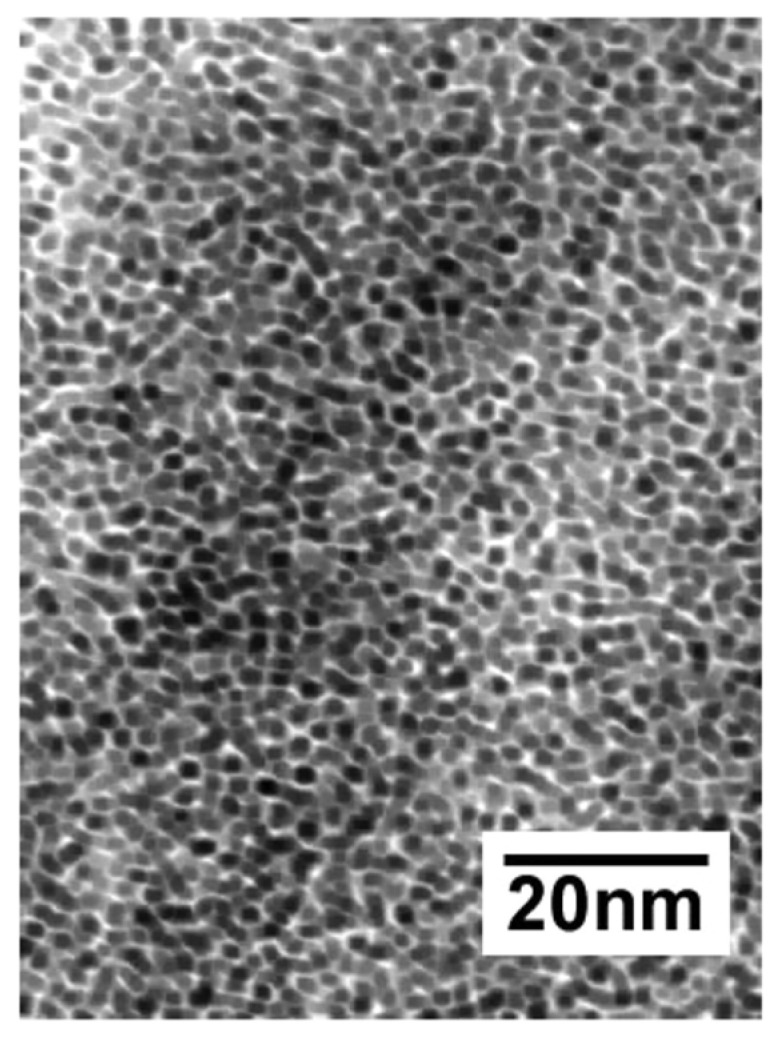
TEM micrograph of a self-assembly thin film of PPh_3_-Pt nanoclusters. Reproduced with permission, Copyright 2002 from American Chemical Society [28].

As an important chemical in many industrial processes and a clean energy source for fuel cells and internal combustion engines, hydrogen is widely used in industrial production and scientific research. A fast and reliable method for detecting H_2_ leakage or accumulation is needed. A great deal of effort has been made to develop H_2_ sensors and to improve their selectivity. We reported a highly selective sensor for detecting H_2_ in air based on TiO_2_/PtO–Pt dual-layer films [28]. The sensor has a dual-layer structure, in which a porous film of surface-oxidized Pt NPs (PtO–Pt) on a glass substrate is covered with a thin film of TiO_2_ nanoparticle. In a typical experiment for preparing the PtO–Pt thin film, a glass or quartz substrate (1 × 1 cm^2^) is immersed in a colloidal solution of PPh_3_-modified Pt NCs (PPh_3_–Pt) in toluene [10] for 48 h. During this time, a uniform self-assembly film of PPh_3_–Pt NCs with a thickness of ca. 100 nm forms on the substrate surface (Figure 10), which is washed with the solvent, dried at 100 °C, and then thermally treated in air at 400 °C to produce the porous PtO–Pt film.

The nanostructured dual-layer films were designed to take advantage of the partial reduction of TiO_2_ with H_2_ upon the catalysis of the PtO–Pt porous film, resulting in an increase in the charge carrier concentration of the TiO_2_ film at low temperatures, which may improve the sensor selectivity to H_2_, since H_2_ is a more actively reductant compared to many reductive gases. The prepared sensor exhibited an excellent selectivity and good sensitivity to H_2_ in air, but it was non-sensitive to CH_4_, NH_3_ and CO, allowing us to perform the semi-quantitative detection of H_2_ content in air in the range from 1% to 10%. Moreover, we also successfully prepared dual-layer structure H_2_ sensors by combining a granular thin film of PtO–Pt with a film of TiO_2_ or SnO_2_ nanoparticles. Both TiO_2_/PtO–Pt and SnO_2_/PtO–Pt exhibited high sensitivity and selectivity to H_2_ in air [29].

Lang et al. reported a catalytic gas sensor for H_2_ detection with high sensitivity, a fast response time and a low power consumption. The catalyst layer has a porous network structure formed by linking unprotected Pt NCs with phenylenediamine, which acts as a ligand [61]. Leghrib et al. fabricated an active layer of sensors assembled with MWCNTs and PVP-modified unprotected Rh NPs. The fabricated sensors were used for detecting NO_2_, C_2_H_4_, CO and C_6_H_6_ at room temperature [187].

Amperometric glucose sensors have been widely developed due to their high practicability for taking glucose measurements. Zhu et al. prepared a Pt-SiO_2_ composite assembled with the unprotected Pt NCs (d_av_ = 2 nm) and the SiO_2_ nanoparticles (d_av_ = 13 nm), which was used as an immobilization carrier of glucose oxidase to fabricate a glucose biosensor with high sensitivity to glucose and good stability [188]. Chen et al. developed a bilayer film composite by electrodepositing the unprotected Pt NCs on a Prussian blue-gold (PB-Au) nanocomposite film, which was used as a support for anchoring the glucose oxidase (GOD). The electrode bearing glucose oxidase was covered with a Nafion film to increase the sensor’s stability. The prepared biosensor performed well, with a fast response time (within 8 s), high sensitivity and a wide linear calibration range for detecting glucose [189].

## 4. Concluding Remarks and Outlook

After more than 20 years of development, significant progress has been made in the synthesis and study of the formation mechanism, particle size and alloy composition regulation, interphase transfer and long-term storage, surface modification, immobilization or encapsulation in porous materials and atomization on the support surface of small-sized unprotected metal nanoclusters synthesized by the alkaline EG method. These metal nanoclusters have played important roles in the construction of efficient catalysts for many chemical and electrochemical reactions and sensing materials, and in the exploration of the structure–function relationship of metal catalysts. Based on some experimental and theoretical research results, the principle of the influence of support, ligands and modifiers in metal nanocluster catalysts on the catalytic properties is proposed in this paper, which will play an important role in understanding the structure–function relationship and improving the catalytic properties of metal catalysts. In this field, there are at least the following challenging scientific and technical issues that deserve attention and further effort:

The firsts are the preparation of structure-controllable catalysts using unprotected metal nanoclusters as building blocks or precursors and the deepening of the understanding of the structure–function relationship of metal catalysts, especially that between the charge distribution of metal nanoclusters at the molecular or atomic scale and the catalytic performance. In situ characterization techniques and theoretical calculations are important means to achieve this goal.

Another one is developing new synthesis or assembly methodologies for creating high-performance metal catalysts based on unprotected metal nanoclusters. Encapsulating metal nanoclusters with designed materials, such as molecular sieve, MOF, COF or ligands on supports is a promising way to construct a delicate chemical environment around the metal nanoclusters to improve the selectivity and stability of the metal catalysts. New preparation methods for highly efficient and stable metal and alloy nanocluster catalysts with good metal loading and metal dispersion properties based on the immobilization of unprotected metal nanoclusters is of significance in the conversion and utilization of renewable energy. This requires an in-depth understanding of the surface chemistry of unprotected metal nanoclusters.

The unprotected metal nanoclusters exhibited some unique chemical properties, including atomization on the support surfaces and the catalytic conversion of the inorganic compounds, which means that they are promising for basic research and applications.

The technologies of regulating the size, structure, composition and morphology of unprotected metal and alloy nanoclusters over a wide range and with high accuracy still need to be further improved.

## Figures and Tables

**Table 1:** Fitting parameters of the Ru-Cl, Ru-O, and Ru–Ru bonds obtained from the Ru *K*-edge EXAFS spectra of the reaction mixture for preparing unprotected Ru nanoclusters. Reproduced with permission, Copyright 2020 Editorial office of Acta Physico-Chimica Sinica [102].

Time/min	T/°C	CN(Ru–Cl)	CN(Ru–O)	CN(Ru–Ru)	R(Ru–Cl)/Å	R(Ru–O)/Å	R(Ru–Ru)/Å
0	27	0.56	6.27		2.40	2.05	
2	48.9		5.65			2.05	
36	100		4.45			2.06	
39	100		3.56	0.21		2.07	2.68
61	100		1.57	2.87		2.12	2.71
62	100			3.58			2.71
120	100			3.83			2.71

**Table 2:** Fitting parameters of the Pt–Cl, Pt–O, and Pt–Pt bonds obtained from the Pt *L*_3_-edge EXAFS spectra of reaction mixture for preparing unprotected Pt nanoclusters. Reproduced with permission, Copyright 2020 Editorial Office of Acta Physico-Chimica Sinica [102].

Time/min	T/℃	Bond	CN	r/Å	ΔE/eV	σ^2^/Å^2^	R/%
0	RT (24.9)	Pt–Cl	4.86	2.31	12.24	0.0027	0.24
7	78.3	Pt–Cl	3.05	2.32	12.22	0.0030	0.71
		Pt–O	1.62	2.01	10.54	0.0009	
9	80.0	Pt–Cl	2.09	2.33	14.97	0.0026	0.61
		Pt–O	2.98	2.05	11.83	0.0051	
10	80.0	Pt–O	1.97	2.13	7.75	0.0037	0.95
		Pt–Pt	3.20	2.74	5.53	0.0046	
11	80.0	Pt–Pt	5.34	2.74	5.53	0.0074	0.45
90	80.0	Pt–Pt	9.09	2.76	8.82	0.0076	1.77

**Table 3:** The average particle size of colloidal nanoparticles in the reaction mixture for preparing Pt nanoclusters measured by TEM and DLS. Reproduced with permission, Copyright 2020 Editorial Office of Acta Physico-Chimica Sinica [102].

Reaction Condition	*d*_av_ (TEM)/nm	*d*_av_ (DLS)/nm
–	3.7	7.1
80 ℃–30 min	2.4	6.3
80 ℃–60 min	1.6	5.9
80 ℃–90 min	1.4	3.7

**Table 4:** The reaction energy barriers calculated via VASP software. Reproduced with permission, Copyright 2022 The Royal Society of Chemistry [55].

Catalyst	Reaction Energy Barriers (eV)		
	CH_2_ + CH_2_ Coupling	CH_2_ Hydrogenation	CH_3_ Hydrogenation
Pt_42_-Ru8	0.31	0.55	0.53
Ru50	1.44	0.85	0.77
Pt_50_	0.78	0.92	0.81

## Data Availability

Not applicable.
